# Concordance of the point-of-care circulating cathodic antigen test for the diagnosis of intestinal schistosomiasis in a low endemicity area

**DOI:** 10.1186/s40249-019-0551-7

**Published:** 2019-05-30

**Authors:** Sergei Rodrigo Magalhães de Sousa, Isabelle Helena Lima Dias, Álvaro Luan Santana Fonseca, Bianca Rodrigues Contente, Joyce Favacho Cardoso Nogueira, Tatyellen Natasha da Costa Oliveira, Stefan Michael Geiger, Martin Johannes Enk

**Affiliations:** 1 Programa de Pós-Graduação Strictu Sensu em Biologia Parasitária na Amazônia da Universidade do Estado do Pará, Tv. Perebebuí, 2623 - Marco, Belém, PA 66087 Brazil; 2 Laboratório de Parasitoses Intestinais, Esquistossomose e Malacologia (LPIEM), Secção de Parasitologia, Instituto Evandro Chagas/SVS/MS, Ananindeua, Pará Brazil; 3 Departamento de Parasitologia, Universidade Federal de Minas Gerais, Belo Horizonte, Minas Gerais Brazil

**Keywords:** *Schistosoma mansoni*, Kato-Katz, POC-CCA, Accuracy, Diagnosis, Low prevalence area

## Abstract

**Background:**

The Kato-Katz technique is recommended worldwide for the diagnosis of intestinal schistosomiasis, detecting parasite eggs in feces of infected people. However, new tests have been developed in order to facilitate diagnosis, e.g. by detection of specific antigens secreted by schistosomes, such as the circulating cathodic antigen (CCA). The aim of this study was to evaluate the performance of the point-of-care circulating cathodic antigen test (POC-CCA) compared to the Kato-Katz technique in a low prevalence area in the Amazon Region, located in the municipality of Primavera, State of Pará, Brazil.

**Methods:**

Positivity rates of the POC-CCA test and the Kato-Katz technique were calculated. The sensitivity, specificity, accuracy and kappa coefficient were determined by comparing both methods. The reference standard was established using 16 Kato-Katz slides, 12 of the first fecal sample, two of the second and two of the third one. The study also included the concordance between POC-CCA results and different numbers and combinations of Kato-Katz slides.

**Results:**

The prevalence of schistosomiasis according to the reference standard or POC-CCA test reached a rate of 9.4% or 23.9%, respectively, among a total of 372 participants. The positivity rates by the Kato-Katz technique increased from 2.4 to 9.4%, according to the increase in the number of slides examined and fecal samples collected. A sensitivity of 55.6%, specificity 76.9%, accuracy 76% and *κ* coefficient of 0.06 was observed by comparing one slide of the first sample and POC-CCA. Comparing 6 slides from three different samples, two slides of each, with POC-CCA resulted in a sensitivity of 58.3%, specificity 78.4%, accuracy 77% and *κ* coefficient of 0.16. Finally, the comparison of 16 slides from three different samples with POC-CCA revealed a sensitivity of 65.7%, specificity 80.4%, accuracy 79%, and *κ* coefficient of 0.27.

**Conclusions:**

The immunochromatographic test has the potential to be an important tool to combat schistosomiasis because of its practicality and applicability but should be applied with caution in low prevalence areas and in programs that aim to eliminate this disease.

**Trial registration:**

CAAE#21824513.9.0000.5091. January 31st, 2014.

**Electronic supplementary material:**

The online version of this article (10.1186/s40249-019-0551-7) contains supplementary material, which is available to authorized users.

## Multilingual abstracts

Please see Additional file 1 for translations of the abstract into the five official working languages of the United Nations.

## Background

Schistosomiasis is a neglected disease of great matter in public health. The World Health Organization (WHO) organized WHA65.21 resolution adopted by endemic countries in World Health Assembly 2012, aimed to intensify the combat of this parasitic disease as a public health problem in endemic countries. The main objective is reducing positivity rate to less than 5.0% [1].

It is estimated that more than 206 million people required preventive treatment in 2016 to schistosomiasis around the world. Schistosomiasis transmission has been reported from 78 countries [2]. More than 1.5 million of these individuals are in risk areas in Brazil [3]. From 2010 to 2016, a total of 6 233 975 exams were performed, achieving endemic areas of Brazil, revealing a total of 273 019 positives detected, with a positivity rate of 4.4% [4].

Currently, Kato-Katz technique is the recommended method for *Schistosoma mansoni* diagnosis [5], a quantitative and qualitative direct test besides low cost and high specificity, often underestimates positivity rates among infected individuals with low parasite burden in low infection rate areas [6–12].

Rapid methods, also called point of care (POC) tests, have been developing to facilitate schistosomiasis diagnostic. These immunochromatographic tests detect the presence of worm antigen in serum or urine, specifically circulating anodic antigen (CAA) and circulating cathodic antigen (CCA), respectively [13]. The advantage of using circulating antigens is the easy manipulation of urine samples in research field and the simple reading of results, not being necessary advanced training [14].

In 2015 WHO recommended the use both methods, POC-CCA and Kato-Katz in order to evaluate and monitor the control programs efficiency in endemic countries if the main goal is to eliminate schistosomiasis as a public health problem. Other methods are still being evaluated, as Helmintex® test that has a great probability to become the “gold standard” to the diagnosis of schistosomiasis [15].

In Tanzania and Kenya, the POC-CCA test was used to identify *S. mansoni* antigen in urines from children, showing higher prevalence rates when compared to the Kato-Katz technique [16]. In Uganda this fast test has also shown better results when compared to two Kato-Katz slides [17]. According to data from Europe, the application of POC-CCA among immigrants of endemic areas is quite encouraged, due to its practicality and high sensitivity [18]. Nowadays, the POC-CCA test is already being used to map *S. mansoni* infections in Africa [19].

Kittur et al. in a systematic review described a direct association between POC-CCA and Kato-Katz test results in areas with prevalence higher than 50% [20]. In areas with prevalence lower than 10.0%, there is not enough evidence to evaluate the performance of the POC-CCA test [21]. In this point of view, this study aims to compare both diagnostic approaches in a low prevalence area, assessing the performance of the rapid test and its possible application in large scale.

## Methods

### Population and study area

This research was performed in the communities of Pedrinha and Canaã, municipalities of Primavera, located in the mesoregion of Northeastern Pará State, distant about 195 km from the capital Belém. The area contains 258.6 km^2^ and a population density of approximately 40 inhabitants/km^2^. The communities are part of the legal Amazon region, with tropical climate and temperatures ranging from 20 °C to 40 °C. It presents climatic conditions where the annual average rainfall varies between 2500 mm and 3000 mm [22].

A total of 422 local residents were invited to participate of this research. Since the epidemiological study involved the whole population, there was no need for a representative sample size calculation. Previous surveys from Brazilian Schistosomiasis Control Program reported that the region is a low prevalence area, presenting positivity rates between 2.0 and 3.0%.

Individuals who did not deliver three fecal and urine samples were excluded from the study. In addition, children less than 2 years old and pregnancy women were also excluded, which led to a reduction of the total population to 372 individuals.

### Collection of biological samples

**Fig. 1:**
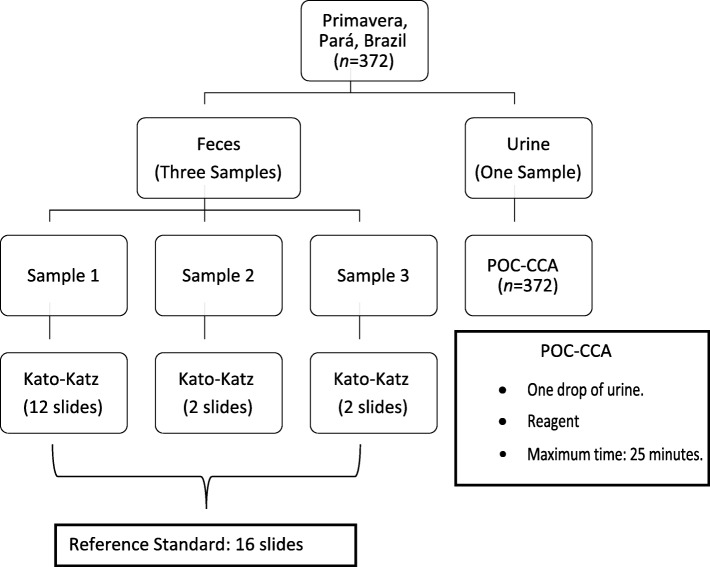
Flowchart of stool and urine samples processing. Legend: POC-CCA: point-of care circulating cathodic antigen

Three fecal samples and three first morning urine samples were collected from the 372 remaining participants on three consecutive days (Fig. 1). For examination, the three fecal samples were processed while only one urine sample was used. The remaining urine samples were frozen and stored in a freezer at the Biobank of the Parasitology Section of the Instituto Evandro Chagas – Belém, Pará, Brazil.

Urine and fecal samples were processed and analyzed according to the protocols of each technique [5, 23]. To ensure quality standards, 10.0% of the slides and rapid tests were independently read by a second technician, as defined by the laboratory’s current standard operational procedures.

The samples were codified and the results of the immunochromatographic test were recorded with sequential numbers per participant in order to preserve anonymity.

## Diagnostic methods

### Kato-Katz

The Kato-Katz slides [5] were prepared using the *Helm Test* Kit*®* (Biomanguinhos, Ministry of Health, Brazil). A total of 16 slides, 12 of the first sample, two of the second one and two of the third fecal sample were used. The 12 slides from the first sample summed up to 500 mg of examined fecal matter. Together with the slides from samples two and three, a total of about 667 mg of feces were analyzed, which, in this case, was established as the reference standard.

**Fig. 2:**
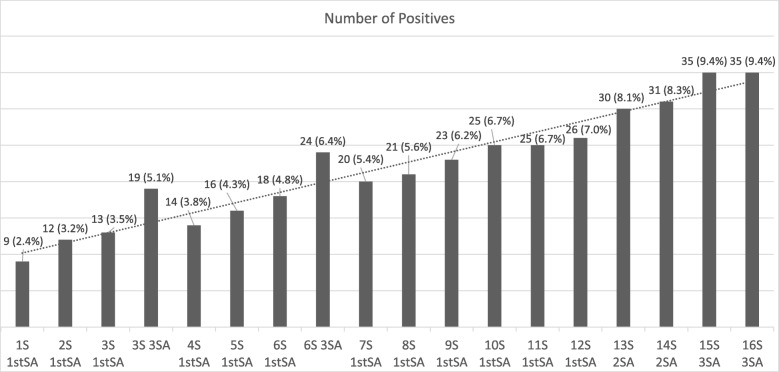
The cumulative prevalence calculated by Kato-Katz technique using combinations of slides and samples. Legend: S: Slide; SA: Sample.

In addition to the reference standard, different random combinations of slides and samples were used to perform the Kato-Katz technique, as described in Fig. 2.

### POC-CCA

The urine-CCA cassette is recommended for the qualitative presumptive detection of an active *Schistosoma* infection, being *S. mansoni* more specific. According to the manual of the producer Rapid Medical Diagnostics, the test may show false negative results in case of low infection levels. In high transmission settings, the sensitivity can reach 100% and in low transmission settings 70.0% [23].

**Fig. 3:**
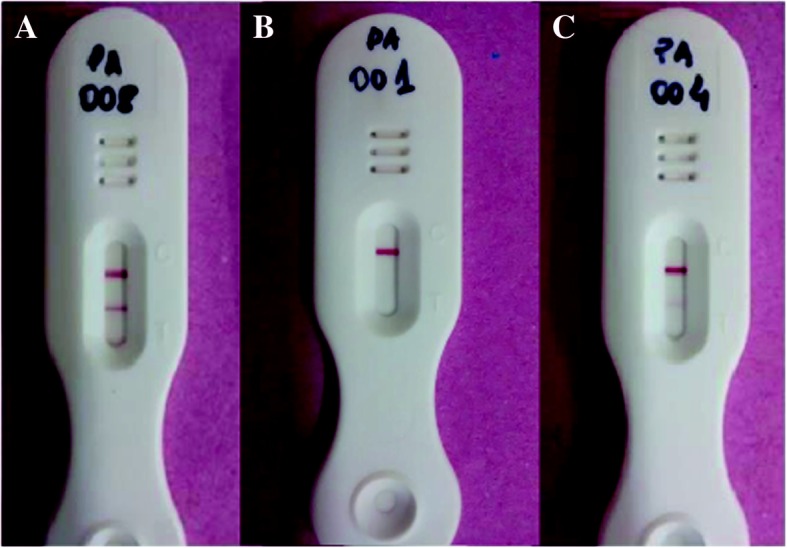
Results of three POC-CCA tests. Legend: **a** Positive, **b** negative and **c** “Trace”

Only one drop of first morning urine was required for the examination. The test result was reported between 20 and 25 min after adding samples and buffer. The blue line to test control should become pink to valid the test; any other color outside this pattern was considered invalid. All the results of the immunochromatographic test were interpreted as positive, considering the development of a second pink line, parallel to the control, otherwise the test was considered negative, according to the manufacturer recommendations (Rapid Medical Diagnostics, South Africa, 2015) [23]. It’s important to note that a weak pink line, classified as ‘trace’ result, was included into the analysis, first as positive and second as negative result, as shown in Fig. 3.

### Statistical analysis

Statistical tests were performed by the online program OpenEpi version 3.01 (Open Source Epidemiologic Statistics for Public Health, available on https://www.openepi.com/Menu/OE_Menu.htm updated in 2013). The topic calculations: paired 2 × 2 tables in a 95% confidence interval (*CI*). The rate of positivity, sensitivity, specificity, positive and negative predictive values and general accuracy were calculated. The kappa index was calculated to evaluate the concordance between the results obtained by the POC-CCA and the Kato-Katz method, following the classification criteria recommended by Landis & Koch (1977) [24]. To observe statistically significant differences, the McNemar test was applied, with results considered significant if *P* ≤ 0.05 and at a confidence interval of 95%.

### Ethical considerations

The study is part of a multicenter project, with participants from Pará, Minas Gerais and Santa Catarina. The project was submitted to the ethics committee and approved with CAAE: 21824513.9.0000.5091. All participants were informed about the objectives and invited to participate voluntarily after signing the Informed Consent Form. All positive individuals according to the reference standard were treated with praziquantel, following the guidelines of the Brazilian Ministry of Health, with 60 mg/kg for children and 50 mg/kg for adults.

## Results

### Positivity rate by Kato-Katz technique

This study consisted of 372 participants, 186 (50.0%) male and 186 (50.0%) female. The positivity rate for schistosomiasis determined by Kato-Katz technique with 16 slides was 35 (9.4%) positive individuals. The positivity rate obtained by the Kato-Katz technique increased from 9 positives (2.4%) to 35 positives (9.4%), depending on the number of slides of the samples examined by participants. Sample diversification detected more positives when compared to results from one sample (Fig. 2).

Regarding to eggs count per gram (EPG) revealed by reference standard, all the individuals shown a low parasitic load. Out of 35 positive individuals, 18 had been shown two EPG. Only a single participant had a parasitic load with 332 EPG. The others 16 infected revealed an individual’s count between three and 47 EPG.

About gender distribution of infection, the number of positives shown a total of 30 males infected, indicating an infection rate of 16.1% and five positive females with an infection rate of 2.7%.

### Positivity rate by POC-CCA test

The presence of infection in children less than 10 years old was only identified by the POC-CCA test. In relation to other age groups, the Kato-Katz technique detected more positive individuals between 21 and 40 years old as described in Table 1.

**Table 1:** Positivity rate in different age groups revealed by the Kato-Katz technique and the rapid urine test (POC-CCA)

Age group	Total Number	Kato-Katz		POC-CCA	
		Number of positives	Positivity rate	Number of positives	Positivity rate
≤ 10	70	0	0	10	14.3%
11–20	82	6	7.3%	6	7.3%
21–30	54	7	13.0%	5	9.2%
31–40	53	8	15.1%	5	9.4%
41–50	48	6	12.5%	22	45.8%
51–60	25	2	8.0%	16	64.0%
61–70	22	4	18.2%	16	72.7%
< 70	18	2	11.1%	9	50.0%

*POC-CCA* point-of-care circulating cathodic antigen

The positivity rate determined by the immunochromatographic POC-CCA test was 23.9% (89 positive individuals). It is important to emphasize that results with faint or poor visualization compared to the control range were considered “Trace”, being classified as positive (Fig. 2).

### Accuracy analysis of the POC-CCA test

Accuracy and matching values of the POC-CCA test results compared to the Kato-Katz technique demonstrated higher sensitivity as the number of slides increased. The sensitivity of 55.6% was observed when compared POC-CCA results with positives detected by one slide of Kato-Katz, however, it is increased to 65.7% when was compared with 16 slides of different samples (Table 2). Statistically significant differences were observed in all results of Kato-Katz slides and samples when compared with the results of the immunochromatographic test, with *P* < 0.0001.

**Table 2:** Concordance (coefficient kappa) and accuracy analysis between Kato-Katz test results obtained by an increasing number of slides and the results of one rapid urine test sample (POC-CCA)

POC-CCA	Kato-Katz 1S 1st AS			Kato-Katz 6S 3SA			Kato-Katz 16S 3SA		
	Positive	Negative	Total	Positive	Negative	Total	Positive	Negative	Total
Positive	5	84	89	14	75	89	23	66	89
Negative	4	279	283	10	273	283	12	271	283
Total	9	363	372	24	348	372	35	337	372
Sensitivity	55.6% (95% *CI*: 26.6–81.1)			58.3% (95% *CI*: 38.8–75.5)			65.7% (95% *CI*: 49.1–79.1)		
Specificity	76.9% (95% *CI*: 72.2–80.9)			78.4% (95% *CI*: 73.8–82.4)			80.4% (95*% CI*: 75.8–84.3)		
PPV	6.0% (95% *CI*: 2.4–12.5)			16.0% (95% *CI*: 9.6–24.6)			26.0% (95% *CI*: 17.9–37.0)		
NPV	98.6% (95% *CI*: 96.4–99.4)			96.5% (95% *CI*: 93.6–98.1)			95.8% (95% *CI*: 92.7–97.6)		
Kappa index	0.06 (95% *CI*: 0.01–0.1)			0.16 (95% *CI*: 0.1–0.2)			0.27 (95% *CI*: 0.2–0.3)		
Accuracy	76.0% (95% *CI*: 74.6–82.8)			77.0% (95% *CI*: 72.6–81.1)			79.0 79.0% (95% *CI*: 74.6–82.8)		

*CI* Confidence interval, *PPV* Positive predictive value, *NPV* Negative predictive value, *S* Slides, *SA* Sample, *1S 1st SA* One slide of the first sample, *6S 3SA* Six slides of three different samples, two slides of each sample, *16S 3SA* 16 slides of three different samples, 12 slides of the first sample, two slides of the second sample and two slides of the third sample

### “Trace” classified as negative results

In the case that the total number of 74 POC-CCA results classified as “Traces” were considered as negative, the number of remaining positives would be reduced to 15 individuals. Table 3 provides detailed information about the positive and negative POC-CCA test results paired with the results of 16 Kato-Katz slides. It is important to note that a total of 17 results out of the 74 “Traces” considered as negative were confirmed as positive by Kato-Katz technique.

**Table 3:** Positive and negative results obtained with the POC-CCA test considering “Traces” as negative results comparing to the Kato-Katz technique, examining 16 slides from three different samples

POC-CCA	Kato-Katz		
	Positive	Negative	Total
Positive	6	9	15
Negative	29	328	357
Total	35	338	372

*POC-CCA* Point-of-care circulating cathodic antigen test

The immunochromatographic test with “Trace” classified as negative revealed sensitivity of 17.1% and specificity of 97.3%. Positive and negative predictive values were 40.0 and 91.9%, respectively. The kappa value was 0.19, classified as weak concordance.

## Discussion

The examination of a Kato-Katz slide is the strategy applied for the diagnosis, treatment of infected individuals and to calculate the prevalence in endemic areas by the Schistosomiasis Control Program [25]. This strategy induced a decrease in the prevalence and intensity of infection over the years, resulting in the difficulty of detecting infections with low parasite burden, since it is known that the technique has failed to detect eggs in these individuals. Consequently, it is necessary to seek for tests with an increased sensitivity, but at reasonable costs [1, 6, 7, 9, 11, 12, 26].

In the scientific community it is well know that detection rate of positives, using the Kato-Katz technique, improves with the increases of the number of slides and stool samples examined [6–10, 26, 27]. Therefore, in this study, 16 slides from three different samples were used as reference test revealing 35 egg-positive individuals. The reference test detected 26 more positives than one Kato-Katz slide, indicating an increase of 74.0% in the detection of positives. One sample with 12 slides detected 26 positives, nine less than compared with the reference standard, resulting in a loss of 25.0% of positives detected. Using different samples, losses of positives were detected when compared to the reference standard (Fig. 2).

The low individual parasitic load found in our study indicates the difficulty in detecting infected individuals, even using 16 slides from three different samples. The more quantity of feces was used, the more egg-positive individuals were detected, as seen in Fig. 2. A coproscopical test with higher sensitivity could be a good strategy to maximize the detection of positives. Oliveira et al. [28] conducted a research combining different parasitological techniques, such as Helmintex® test, Saline gradient and Kato-Katz technique, to evaluate POC-CCA test. Their study revealed an underestimation of the “real” *S. mansoni* prevalence and a maximization of detection of egg-positive individuals with the combination of methods and increased diagnostic efforts.

When compared to the Kato-Katz technique, POC-CCA rapid test has shown similar results, especially when applied in endemic areas with prevalence above 50.0%. This result encourages the application of this rapid test for the combat against schistosomiasis, because it is sufficiently efficient, has relatively low costs and easy and fast application. However, in areas with lower prevalence, the results of the immunochromatographic test tend to show higher values than the coprology test, reaching prevalence 2.5 times higher, as seen in the study of Colley et al. and Kittur et al. [19, 20].

The present study revealed that in low prevalence areas, such as the municipality of Primavera, state of Pará, the rapid test has a positivity rate of 23.9%. The prevalence of the Kato-Katz technique by the reference standard is 9.4%, that is, the POC-CCA has a detection rate 2.5 times higher. In comparison with only one analyzed Kato-Katz slide, we even reached positivity rates through POC-CCA, which were up to 10 times higher. This data is often seen in studies which involve an evaluation of POC-CCA test even in different endemic setting [19, 20].

Studies in endemic regions with a high positivity rate have demonstrated a good performance of the immunochromatographic test when compared to Kato-Katz technique. Adriko et al. [17] evaluated the CCA in different locations in Uganda and found a sensitivity of 92.0% and a specificity of 50.0% of the POC-CCA test in an area with a high positivity rate (55.0%) compared to 6 slides from three different samples of Kato-Katz. Using the same number of slides and samples in an area with a lower positivity rate (13.0%), the sensitivity of the POC-CCA test decreases to 75.0%, however the specificity increases 55.0%. Shane et al. [29] also observed a sensitivity of 94.2% and specificity of 59.4% of the rapid test when compared to 6 Kato-Katz slides from three different samples in a western region of Kenya, with a positivity rate of the coprology test of 38.8%.

In this study, the sensitivity of the rapid test, when compared with six slides of three different samples, was 58.3%. The decrease in sensitivity can be explained by the presence of “false negatives”, with higher frequency in regions presenting individuals with low parasitic load [26]. However, Ferreira et al. [30] detected a sensitivity rate of 55.5% when POC-CCA was compared to 6 slides from three different samples. When using one Kato-Katz slide, the sensitivity of POC-CCA was 64.3%. So, there was a decrease in sensitivity with increasing numbers of slides and samples, differently from that observed in our study (Table 2).

Concerning the distribution of positive and negative POC-CCA by age group, the data in Table 1 highlight that 10 positive individuals (younger than 10 years) are found, unlike what was observed in the results of Kato-Katz. The most positive age groups detected by the immunochromatographic test were from 41 to 70 years of age, but the higher positivity rate was 72.7% in individuals aged from 61 to 70 years old. In this context, the rapid test demonstrates that individuals over 41 years of age are most affected by the disease, contrasting with the Kato-Katz technique that indicates individuals over 61 years of age as the most affected.

In order to improve the sensitivity of POC-CCA test, Grenfell et al. [31] modified the urine processing and concentrated the circulating cathodic antigen by combining the test with urine filtration-concentration method. This research revealed a new way to understand “Trace” results. Nevertheless, more extensive studies in other areas with low individual parasite load are necessary to confirm these findings.

In the present study, the POC-CCA showed a higher sensitivity (65.7%), higher kappa value (0.27), characterized as “fair”, and better accuracy when compared to the reference standard. These results demonstrate the ability of the immunochromatographic test to detect positives also diagnosed by the reference test, especially in low prevalence areas. It’s important to emphasize the same pattern had been found by Siqueira et al. [32] in relation to a progressive sensitivity and kappa value, however, in a high prevalence area.

Concerning the difficult visualization of some results from the immunochromatographic test, a classification characterized as “Trace” has been established, especially among individuals with low parasite load. However, some studies suggest considering “Trace” as positive, which led to the use of “weak positives” as synonyms by several authors [16, 33, 34]. In our study, the “Trace” was considered as positive result, as originally recommended by the manufacturer and the test kit imported from South Africa. Although other studies also use “Trace” results to observe intensity of infection [30, 31, 34].

However, recent publications propagate that “Traces” should be classified as negative [31, 35]. In our study, transforming 74 “Trace” results into negatives and comparing to the reference standard, a decrease in sensitivity of the POC-CCA test from 65.7% (“Trace” as positive) to 17.1% (“Trace” as negative) was found. In addition, 17 individuals diagnosed as positives by Kato-Katz technique were missed.

It is important to emphasize that the comparison of the reference standard with POC-CCA results considering “Trace” as positive; a total number of 23 true positives were detected by POC-CCA test. In contrast, considering “Trace” as negatives, a total of six true positives were identified by POC-CCA test. That means, 17 true positives would be lost. Besides that, the 23 true positives diagnosed by the immunochromatographic test were similar to the results of nine Kato-Katz slides from the same fecal sample or six slides of three different samples, which indicates, according to the data of this study, a higher sensitivity of the POC-CCA when compared with two Kato-Katz slides as currently used by the governmental control program.

These findings corroborate another study, considering “Trace” results either as positive or as negative, in which “Trace” classified as positive, revealed a better performance of the test, detecting 108 infected individuals out of a population of 228 participants, with a POC-CCA test sensitivity of 64.9%, specificity of 69.2%, and kappa value of 0.34. Therefore, when “Trace” was classified as negative, the sensitivity revealed low percentage of 26.8% and kappa value of 0.25 [30].

Regarding easy applicability of POC-CCA under field conditions when compared to Kato-Katz technique, a great advantage of POC-CCA is observed. When using 16 Kato-Katz slides from a total of 372 participants, 5952 slides were prepared and read for detection of *S. mansoni* eggs. With 8 h of daily work, 60 slides can be read within this period by a technician, taking three and a half months of work to detect *S. mansoni* infection from all participants. When applying the rapid test point-of-care circulating cathodic antigen in a urine sample, 25 min are required per patient to determine the outcome. A technician performed 300 tests a day, being 40 tests per hour. So, more than 80.0% of participants had been examined for schistosomiasis using the POC-CCA test in just 1 day.

In this work, we present results that should be carefully observed, since they may contribute to a better understanding in the combat against schistosomiasis in areas with low infection rates. The POC-CCA accuracy values demonstrate low sensitivity of the test in a low prevalence area, which is the focus of this study. In this initial analysis, these data do not show a satisfactory result in the use of the test as an auxiliary tool in the control of schistosomiasis in areas with low infection rates.

The presence of false negatives is an important issue that should be debated. There are a lot of reasons which could affect the detection of CCA in human urine. The most reasonable explanation mentioned in Rapid Medical Diagnostics [23] is the need about 50 worms or more to CCA can be detected. However, more methodologic studies involving CCA detection among patients with very low worm burden should be carried out to elucidate this issue.

Nevertheless, if the POC-CCA test was applied in the studied population, 89 positives would be detected. Of these, 66 will be unnecessarily treated, since the technique of Kato-Katz with 16 slides indicates absence of eggs in these individuals previously analyzed. Though, 12 (34.3%) positives escape the treatment by the immunochromatographic test (Table 2). However, if a single slide of Kato-Katz technique, which is widely used to control intestinal schistosomiasis, had been applied, the data would reveal that 26 positives won’t be detected, representing 74.3% of infected won’t be treated (Fig. 2).

## Conclusions

The best strategy which involves speed, low cost and better performance in detecting positives to schistosomiasis within the endemic area should be considered. This approach also involves the evaluation of risks and benefits administering praziquantel to individuals without infection detected by the POC-CCA test or in the loss of infected individuals not detected by a single slide of Kato-Katz technique. This study demonstrates that the use of POC-CCA test has similar detection of infected individuals when compare with 9 slides from single sample or 6 slides from 3 different samples. The increase of the sensitivity has shown a better performance from POC-CCA test according to the increase of amount of feces analyzed. Our results show poorer CCA test performance which can be justify by the very low individual egg count per gram. The impact of other issues such as urinary infection and co-infection with other helminthes on the test performance should be investigated in future studies. Thus, the immunochromatographic test has the potential to be an important tool to combat schistosomiasis because of its practicality and applicability but should be applied with caution in low prevalence areas and in programs that aim to eradicate this disease as soon as possible.

## Additional file

Additional file 1: Multilingual abstracts in the five official working languages of the United Nations. (PDF 560 kb)

## Abbreviations

CAA: Circulating anodic antigen
CCA: Circulating cathodic antigen
*CI*: Confidence Interval
EPG: Egg count per gram
POC: Point-of-care
S: Slide
SA: Sample
WHO: World Health Organization
