# Supplementary material for: Concordance of the point-of-care circulating cathodic antigen test for the diagnosis of intestinal schistosomiasis in a low endemicity area
Source: Infect Dis Poverty. 2019 May 30;8:37. doi: 10.1186/s40249-019-0551-7 (PMC6542115; doi:10.1186/s40249-019-0551-7)
Supplement: Supplementary file 1 — Multilingual abstracts in the five official working languages of the United Nations. (PDF 560 kb) [file 40249_2019_551_MOESM1_ESM.pdf]

Translation of the abstract into the five official working languages of the United Nations

مدى توافق وجهة النظر الطبية التي تُرجِح استخدام اختبار المستضد الكاثودي لتشخيص البلهارسيا المعوية في مناطق محدودة العدوى

المؤلفون:-

Sergei Rodrigo Magalhães de Sousa, Isabelle Helena Lima Dias, Álvaro Luan Santana Fonseca, Bianca Rodrigues Contente, Joyce Favacho Cardoso Nogueira, Tatyellen Natasha da Costa Oliveira, Stefan Michael Geiger, Martin Johannes Enk

الملخص:-

اولًا، الخلفية العلمية والمعلومات الأساسية:-

تعتبر تقنية كاتو- كاتز من التقنيات المقترحة والموصى بها لتشخيص البلهارسيا المعوية، حيث تقوم برصد بويضات الطفيل في براز المرضى، وبالرغم من ذلك تم تطوير وسائل واختبارات حديثة لتسهيل التشخيص، على سبيل المثال، رصد مُستضدات (antigens) خاصة بالبلهارسيا ومنها المُستضد الكاثودي المتداول (CCA).

والهدف من هذه الدراسة هو تقييم أداء وجهة النظر الطبية الموصية باستخدام اختبار المُستضد الكاثودي المتداول (POC-CCA)، مقارنة بتقنية الكاتو- كاتز في منطقة محدودة الانتشار في الأمازون الواقعة في بلدية بريمافيرا بولاية بارا بالبرازيل.

ثانيًا، منهجية البحث:-

تم حساب معدل النتائج الإيجابية لكل من اختبار المُستضد الكاثودي المتداول وتقنية الكاتو -كاتز. كما تم تحديد معدل الحساسية والتخصص والدقة ومُعَامِل كابا بمقارنة كل من الطريقتين، وتم تحديد المقياس المرجعي باستخدام ست عشرة شرائح من الكاتو -كاتز واِثْنَتي عشرة عينة من البراز من العينات الأولية، بالإضافة إلى عينتين من كل من عينات البراز الثانية والثالثة، كما احتوت الدراسة على مدى التوافق بين نتائج اختبار المُستضد الكاثودي المتداول والأرقام المختلفة وتشكيلات من شرائح الكاتو- كاتز.

ثالثًا، النتائج -:

وصل انتشار البلهارسيا طبقًا للمقياس المرجعي واختبار المُستضد الكاثودي المتداول إلى 9.4% أو 23.9% على التوالي ضمن 372 مشارك، ولقد زادت معدل النتائج الايجابية لتقنية كاتو -كاتز من 2.4% إلى 9.4% طبقًا للزيادة في عدد الشرائح التي تم تفحصها وعدد عينات البراز المُجمعة، وقد لوحظ 55.6% حساسية و 76.9% تخصص و 76% دقة و 0.06 مُعَامِل كابا عند مقارنة شريحة واحدة من العينة الأولى باختبار المُستضد الكاثودي المتداول. وبمقارنة 6 شرائح من ثلاث عينات مختلفة، أي شريحتين من كل واحدة، مع اختبار المُستضد الكاثودي المتداول نتج 58.3% حساسية و 78.4% تخصص و77% دقة و 0.16 مُعَامِل كابا، وأخيرًا، مقارنة ست عشرة شريحة من ثلاثة عينات مختلفة مع اختبار المُستضد الكاثودي المتداول أنتج 65.7% حساسية و 80.4% تخصص و 79% دقة و 0.27 مُعَامِل كابا.

رابعًا، الاستنتاجات:-

اختبار المناعة الكروماتوجرافي له القدرة ليكون أداة هامة لمحاربة البلهارسيا؛ وذلك يرجع لعمليته وسهولة تطبيقه، ولكن يجب تطبيقه بحرص في المناطق ذات الانتشار المحدود، كما يجب تطبيقه في برامج التي تهدف إلى القضاء على المرض.

Translated from English version into Arabic by Islam Mayhoub, proofread by Sondos Hany, through

TRANSLATORS
WITHOUT BORDERS

在低流行区应用即时循环阴极抗原检测和改良加藤法诊断肠血吸虫病的一致性

Sergei Rodrigo Magalhães de Sousa, Isabelle Helena Lima Dias, Álvaro Luan Santana Fonseca, Bianca Rodrigues Contente, Joyce Favacho Cardoso Nogueira, Tatyellen Natasha da Costa Oliveira, Stefan Michael Geiger, Martin Johannes Enk

摘要

引言：改良加藤法（Kato-Katz）是世界范围内被推荐用于诊断肠血吸虫病，检测感染者粪便中的寄生虫虫卵的方法。然而，为了便于诊断，近期开发了一些新的检测方法，例如通过检测血吸虫分泌的特定抗原如循环阴极抗原(CCA)的检测方法。本研究的目的是在巴西巴拉州 Primavera 市亚马逊地区的血吸虫病低流行区评估即时循环阴极抗原检测(POC-CCA)的性能，其诊断结果与 Kato-Katz 技术作比较。

方法：采用 POC-CCA 法和 Kato-Katz 法检测样本，计算阳性率。比较两种方法的敏感性、特异性、准确性和 kappa 系数。使用 16 张 Kato-Katz 载玻片、12 张第一粪样、2 张第二粪样和 2 张第三粪样建立参考标准。本研究还包括 POC-CCA 与不同 Kato-Katz 玻片数量和组合的诊断结果一致性。

结果：372 名参试者中，参考标准和 POC-CCA 检验血吸虫病患病率分别为 9.4%和 23.9%。随着检查的玻片

和收集的粪便样本的数量增加，Kato-Katz 法的阳性率从 2.4%升至 9.4%。第一粪样的一张玻片和 POC-CCA 比较，敏感性为 55.6%，特异性为 76.9%，准确性为 76%和 κ 系数为 0.06。比较 3 个不同的样本的 6 张玻片，所有样本的两张玻片与 POC-CCA，敏感性为 58.3%，特异性为 78.4%，准确性为 77%和 κ 系数为 0.16。最终，3 个不同粪样 16 张玻片与 POC-CCA 相比，敏感性为 65.7%，特异性为 80.4%，准确性为 79%， κ 系数为 0.27。
结论：由于免疫层析检测的实用性和适用性，该方法有可能成为对抗血吸虫病的重要工具，但在低流行地区和旨在消除血吸虫病的项目中应谨慎使用。

Translated from English version into Chinese by Xin-Yu Feng, edited by Pin Yang

Concordance de la recherche de l'antigène cathodique circulant sur le lieu des soins pour le diagnostic de la schistosomiase intestinale dans une zone de faible endémicité

Sergei Rodrigo Magalhães de Sousa, Isabelle Helena Lima Dias, Álvaro Luan Santana Fonseca, Bianca Rodrigues Contente, Joyce Favacho Cardoso Nogueira, Tatyellen Natasha da Costa Oliveira, Stefan Michael Geiger, Martin Johannes Enk

Résumé
Contexte: La technique de Kato-Katz est recommandée dans le monde entier pour le diagnostic de la schistosomiase intestinale, consistant à détecter les œufs de parasites dans les selles des personnes infectées. De nouvelles méthodes ont cependant été émises au point pour faciliter le diagnostic, par exemple la détection d'antigènes spécifiques sécrétés par les schistosomes, tels que l'antigène cathodique circulant (ACC). Le but de notre étude était d'évaluer les performances du test de recherche de l'antigène cathodique circulant sur le lieu des soin (ACC-POC) par rapport à la technique Kato-Katz dans une zone de faible prévalence située dans la municipalité amazonienne de Primavera, État de Pará, au Brésil.
Méthodes: Les taux de positivité avec le test ACC-POC et la méthode Kato-Katz ont été calculés. La sensibilité, la précision, la spécificité et le coefficient kappa ont été déterminés en comparant les deux méthodes. La référence a été définie en utilisant 16 lames Kato-Katz : 12 du premier échantillon de selles, 2 du deuxième et 2 du troisième. L'étude incluait également la concordance entre les résultats d'ACC-POC et différents nombres et combinaisons de lames Kato-Katz.
Résultats: La prévalence de la schistosomiase atteignait 9,4 % selon la référence ou 23,9 % selon l'ACC-POC sur un total de 372 participants. Le taux de positivité avec la technique Kato-Katz est passé de 2,4 % à 9,4 % avec l'augmentation du nombre de lames examinées et d'échantillons de selles recueillis. La comparaison d'une lame du premier échantillon et de l'ACC-POC a donné une sensibilité de 55,6 %, une spécificité de 76,9 %, une précision de 76 % et un coefficient κ de 0,06. La comparaison de 6 lames de 3 échantillons différents (2 lames de chaque) avec l'ACC-POC a donné une sensibilité de 58,3 %, une spécificité de 78,4 %, une précision de 77 % et un coefficient κ de 0,16. Enfin, la comparaison de 16 lames de 3 échantillons différents avec l'ACC-POC a donné une sensibilité de 65,7 %, une spécificité de 80,4 %, une précision de 79 % et un coefficient κ de 0,27.
Conclusions: Pratique et réalisable, le test immunochromatographique a le potentiel de devenir un outil important dans la lutte contre la schistosomiase. Toutefois, il doit être employé avec discernement dans les zones de faible prévalence et dans les programmes visant l'élimination de la maladie.

Translated from English version into French by Suzanne Assenat, proofread by Ahmad Dabaghzadeh, through

TRANSLATORS
WITHOUT BORDERS

Согласованность результатов тестирования на циркулирующий катодный антиген по месту лечения при диагностике кишечного шистосомоза в регионах с низкой эндемичностью

Серхей Родриго Магальяэс де Соуса, Изабель Елена Лима Диас, Альваро Луан Сантана Фонсека, Бьянка Родгигес Контенте, Джойс Фавачо Кардосо Ногуэйро, Татьельен Наташа да Коста Оливейра, Штефан Михаэль Гейгер, Мартин Йоханнес Энк

Реферат
Предпосылки: Использование метода Като-Катца рекомендовано по всему миру для диагностики кишечного шистосомоза путем обнаружения яиц гельминтов в фекалиях зараженных людей. Однако, для облегчения диагностирования были разработаны новые тесты, например, путем обнаружения специфических антигенов, являющихся секретом таких шистосом, как циркулирующий катодный антиген (ЦКА). Цель данного исследования — провести оценку эффективности тестирования на циркулирующий катодный антиген по месту лечения (ЦКА) по сравнению с методом Като-Катца в области с низкой эндемичностью в регионе Амазонии, расположенном в муниципалитете Примавера, штат Пара, Бразилии.
Методы: Были подсчитаны положительные результаты теста на ЦКА по месту лечения и методом Като-Катца. При сравнении обоих методов определяли показатели чувствительности, специфичности, точности и коэффициент каппа. Эталонный стандарт был установлен при помощи исследования 16 препаратов, приготовленных по методу Като-Катца, 12 из которых — первая проба кала, два — вторая и три — третья. В данном исследовании также рассматривалась согласованность между результатами тестирования на ЦКА по месту лечения и различным количеством и комбинациями препаратов по методу Като-Катца.
Результаты: Распространенность шистосомоза согласно эталонному стандарту или результатам теста на ЦКА по месту лечения составила 9,4% и 23,9%, соответственно, в общей сложности у 372 участников. Положительные результаты теста методом Като-Катца возрастали с 2,4% до 9,4% при увеличении количества исследуемых препаратов и собранных образцов кала. При сравнении одного препарата первого образца и теста на ЦКА по месту лечения показатель чувствительности составил 55,6%, специфичности — 76,9%, точности — 76% и коэффициент *каппа* — 0,06. При сравнении 6 препаратов из трех разных образцов, по два препарата каждого образца, результаты теста на ЦКА по месту лечения выявили, что чувствительность составила 58,3%, специфичность — 78,4%, точность — 77% и коэффициент *каппа* — 0,16. Наконец, при сравнении 16 препаратов трех различных образцов теста на ЦКА по месту лечения было выявлено, что чувствительность составила — 65,7%, специфичность — 80,4%, точность — 79% и коэффициент *каппа* — 0,27.
Выводы: Иммунохроматографический анализ может быть важным инструментом при борьбе с шистосомозом вследствие его практичности и применимости, но его следует применять с осторожностью в регионах с низкой эндемичностью и в рамках программ, целью которых является ликвидация этой болезни.

Translated from English version into Russian by Veronika Demeshchik, proofread by Alexander Somin, through

TRANSLATORS WITHOUT BORDERS

Concordancia de la prueba POC-CCA para el diagnóstico de esquistosomiasis intestinal en una zona de baja endemicidad

Sergei Rodrigo Magalhães de Sousa, Isabelle Helena Lima Dias, Álvaro Luan Santana Fonseca, Bianca Rodrigues Contente, Joyce Favacho Cardoso Nogueira, Tatyellen Natasha da Costa Oliveira, Stefan Michael Geiger, Martin Johannes Enk

Resumen
Introducción: La técnica Kato-Katz se recomienda en todo el mundo para el diagnóstico de esquistosomiasis intestinal, la detección de huevos de parásitos en las heces de las personas infectadas. No obstante, se han desarrollado nuevas pruebas para facilitar el diagnóstico; por ejemplo por detección de antígenos específicos, tales como el CCA (Circulating Cathodic Antigen, en inglés), secretados por esquistosomas. El objetivo de este estudio consistió en evaluar el desempeño de la prueba de POC-CCA (Point-Of-Care Circulating Cathodic Antigen, en inglés) y compararalo con el de la técnica Kato-Katz en una zona de baja prevalencia en la región del Amazonas, en el municipio de Primavera, estado de Pará, Brasil.
Metodología: Se calcularon los índices de positividad de la prueba POC-CCA y de la técnica Kato-Katz. Al comparar ambos métodos, se determinaron los coeficientes de sensibilidad, de especificidad, de exactitud y el coeficiente kappa. Se estableció un parámetro de referencia utilizando 16 láminas Kato-Katz, de las cuales 12 eran de la primer muestra fecal, 2 de la segunda y 2 de la tercera. También se tomó en cuenta la concordancia entre los resultados de la prueba POC-CCA y diferentes números y combinaciones de las láminas Kato-Katz.

Resultados: De un total de 372 participantes, la prevalencia de esquistosomiasis alcanzó un índice de 9,4% o 23,9% conforme al parámetro de referencia o a la prueba POC-CCA, respectivamente. Los índices de positividad de la técnica Kato-Katz aumentaron del 2,4% al 9,4%, siguiendo el aumento en el número de láminas examinadas y las muestras fecales recogidas. Al comparar el POC-CCA con una lámina de la primer muestra, se observó una sensibilidad del 55,6%, una especificidad del 76,9%, una exactitud del 76% y un coeficiente κ de 0,06. Luego, al comparar 6 láminas de tres muestras diferentes, dos láminas de cada una, con el POC-CCA se obtuvo como resultado una sensibilidad del 58,3%, una especificidad del 78,4%, una exactitud del 77% y un coeficiente de κ de 0,16. Finalmente, la comparación de 16 láminas de tres muestras diferentes con el POC-CCA reveló una sensibilidad del 65,7%, una especificidad del 80,4%, una exactitud del 79% y un coeficiente de κ de 0,27.
Conclusiones: La prueba de inmunocromatografía posee el potencial de una herramienta importante para combatir la esquistosomiasis por su practicidad y utilidad. Sin embargo, se debe utilizar con prudencia en las zonas de baja prevalencia y en los programas que busquen eliminar esta enfermedad.

Translated from English version into Spanish by Melanie Lahoz, proofread by Natalia Victoria Gómez, through

TRANSLATORS
WITHOUT BORDERS
